# Eocene Diversification of Crown Group Rails (Aves: Gruiformes: Rallidae)

**DOI:** 10.1371/journal.pone.0109635

**Published:** 2014-10-07

**Authors:** Juan C. García–R, Gillian C. Gibb, Steve A. Trewick

**Affiliations:** Phoenix Lab, Ecology Group, Institute of Agriculture and Environment, Massey University, Palmerston North, New Zealand; University of Florence, Italy

## Abstract

Central to our understanding of the timing of bird evolution is debate about an apparent conflict between fossil and molecular data. A deep age for higher level taxa within Neoaves is evident from molecular analyses but much remains to be learned about the age of diversification in modern bird families and their evolutionary ecology. In order to better understand the timing and pattern of diversification within the family Rallidae we used a relaxed molecular clock, fossil calibrations, and complete mitochondrial genomes from a range of rallid species analysed in a Bayesian framework. The estimated time of origin of Rallidae is Eocene, about 40.5 Mya, with evidence of intrafamiliar diversification from the Late Eocene to the Miocene. This timing is older than previously suggested for crown group Rallidae, but fossil calibrations, extent of taxon sampling and substantial sequence data give it credence. We note that fossils of Eocene age tentatively assigned to Rallidae are consistent with our findings. Compared to available studies of other bird lineages, the rail clade is old and supports an inference of deep ancestry of ground-dwelling habits among Neoaves.

## Introduction

Hypotheses favouring Cenozoic diversification of modern bird orders after the Cretaceous-Palaeogene (K–Pg) mass extinction, inferred from the scarcity of Cretaceous fossils [1]–[3], have been rejected by analyses indicating the origin of several lineages during the Cretaceous [4]–[9]. This conclusion derives from calibration of molecular clocks [10], [11], using ever increasing genetic data, and is supported by a rising number of Cretaceous fossils [12]–[14]. Although there is some uncertainty about phylogenetic placement, most adequate, confident and diagnostic neognath fossil material found from the early Paleogene suggests an extensive diversification of neognaths during the late Cretaceous [15]–[17]. Analyses using mitochondrial and nuclear data largely agree in terms of the age of the lineages leading to the main orders of modern birds [18]–[21], but see [22]. Studies are nevertheless sensitive to calibration and dating tools [23], and a persistent difficulty is the influence of missing lineages on inferences regarding the timing of modern diversity [24], [25].

In particular, studies that use data from single species to represent clades suffer from uncertain node age and placement [26], [27], and are not informative about crown group ages. For example, recent analysis of genetic data for Palaeognathae have shed light on the ancestry of moa (Dinornithiformes) by supporting the flying South American tinamous as sister to moa within the Struthioniformes [28], [29]. This finding supports a hypothesis of flying ancestors for modern day flightless Struthioniformes [30], [31] contrary to the assumed flightless ancestry of the entire group. Separate studies support a recent radiation of moa [32], [33] with their origin occurring only 5 million years ago (Pliocene). This estimated time leaves considerable disparity in the assumption that the common ancestor of species of moa was itself flightless [34]. Similarly, raptors or birds of prey are not monophyletic although they share a primary reliance on carnivory, either by scavenging or by capture of prey, and a number of associated functional niches [25], [35]–[37]. These convergent lifestyle specializations of falcons, hawks and eagles indicate a possible early group of raptors (late-Cretaceous) from which a variety of other carnivore groups have adapted to more aquatic lifestyles [35], [37].

This stem versus crown age problem is common to many phylogenetic studies seeking to identify the timing of diversification within Neoaves, whether associated with location, ecology or taxonomy. Mitochondrial genes and genomes have proved valuable in avian evolutionary studies providing the means to address questions about the placement and recognition of clades within the avian phylogeny and their likely time of diversification [8], [11], [20], [37]–[40]. These studies have mainly focused at the order level, whilst studies at family level are scarce. Recent analysis of mitochondrial DNA (mtDNA) genomes suggests that at least 30 major orders of Neoaves originated in the Cretaceous and survive to the present, but no representatives of families within Gruiformes were included [8].

**Figure 1:**
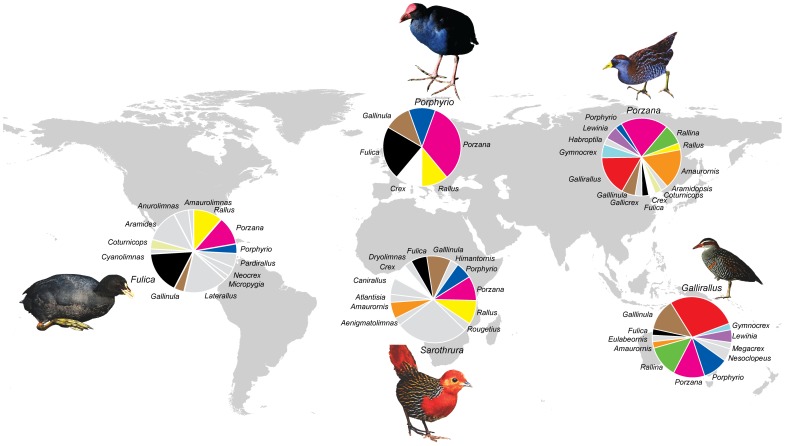
Proportional diversity of genera in Rallidae by geographic regions (Continents). Genera endemic to each continent are in gray. Genera common to more than one region are indicated by identical colour in each pie chart. Taxonomic treatment follows Taylor (1998) and Clements (2012), but we note several lines of evidence place *Sarothrura* outside the family (see Fain et al. 2007 and Hackett et al. 2008).

The rail family (Aves: Rallidae) is globally distributed and extant species occupy niches associated with terrestrial and freshwater habitats. Among these predominantly ground dwelling and foraging birds are a high frequency of flightless species [41], [42]. The ecology and geographic origins of modern rails are almost entirely unknown but most of the putatively “primitive” species, as well as several distinctive genera, inhabit forests of the Old World tropics [42]. Fewer genera are found in the New World, and most of these have been interpreted as being derived from an Old World stem [42]. Some genera (e.g. *Rallus* and *Fulica*) appear to have specialized and radiated in the Americas before reinvading the Old World [42]. There are 135–143 currently recognized species within 33–40 genera [43]–[45], only four of which (*Porzana, Porphyrio, Gallinula*, and *Fulica*) have a worldwide distribution (Figure 1). *Porzana*, as currently recognized, is the most speciose genus in the family (11.9% of rails are currently classified as *Porzana*), followed by *Gallirallus* (11.2%), *Fulica* (7.7%), and *Gallinula, Laterallus, Rallus, Amaurornis* and *Sarothrura* (6.3% each). Molecular analysis shows that *Sarothrura* belongs to a separate lineage from Rallidae, Sarothruridae, and more closely related to the family Heliornithidae [36], [46]–[48], but current taxonomy and bird lists still retain this lineage within Rallidae [43], [44]. Most of the diversity of *Porzana* and *Gallirallus* is found in Asia and Oceania (Figure 1). Asia also contains three endemic genera (*Aramidopsis, Habroptila* and *Gallicrex*) as does Oceania (*Nesoclopeus, Eulabeornis* and *Megacrex*). All of those genera except *Nesoclopeus* are monotypic. Africa has 16 endemic species in seven endemic genera (including *Atlantisia* in the South Atlantic islands), 54 species occur in America, including 27 in eight endemic genera and six of the nine *Rallus* species, while only nine species occur naturally in Europe.

Spatial, morphological and current phylogenetic information suggests that rails may be are old within the Neoaves. Analyses indicate a deep placement of the lineage but the inference is based on limited species representatives and short DNA sequences, which requires better resolution [9], [36], [46], [49]–[51]. In the present study, we use complete mitochondrial genomes to assess temporal diversification within Rallidae. We further compare our estimates with those available for other extant bird lineages in order to shed light on biogeographic/spatial patterns operating in diversification within Neoaves.

## Materials and Methods

### Ethics statement

Museum tissue samples representing *Eulabeornis castaneoventris* (Australian National Wildlife Collection, ANWC50493), *Fulica atra* (Australian National Wildlife Collection, ANWC50980), *Gallirallus philippensis* (Australian National Wildlife Collection, ANWC32326) and *Heliornis fulica* (Museu de Zoologia da Universidade de São Paulo, MZUSP79862) were imported into New Zealand under Massey University guidelines for importation of nonviable animal specimens. Tissue samples representing *Gallirallus australis* and *Porphyrio porphyrio* were collected from road kill animals by the Department of Conservation NZ staff without repository institution, and *Lewinia muelleri* blood sample was taken from a wild caught specimen, which was released at the site of capture, under a permit from the Department of Conservation NZ and ethics committee approval from Department of Conservation Institutional Animal Care and Use Committee (IACUC). This research does not require ethics committee approval as no animal was sacrificed, and there was no animal husbandry, experimentation or welfare concerns. Mitochondrial DNA genomes have been submitted to GenBank: accession numbers KF644581–84; KF701060–62.

### Sampling

The data set compiled new assembled mitochondrial genomes of six species within Rallidae and one species within Heliornithidae plus five published rail mitochondrial genomes. To maximize lineage diversity we selected species using available geographic ecological and phylogenetic information. We include three widespread and flying representatives associated with wetland and grassland areas: *Fulica atra* (common coot; KF644582), *Gallinula chloropus* (common moorhen; HQ896036), and *Porphyrio porphyrio* (purple swamphen; KF701062). *Coturnicops exquisitus* (Swinhoe’s rail; NC012143) found in wetlands and *Rallina eurizonoides* (slaty-legged crake; NC012142) inhabiting forests are both volant species present in Asia. *Gallirallus philippensis* (banded rail; KF701061) is distributed in Asia and Oceania, and *Eulabeornis castaneoventris* (chestnut rail; KF644583) in Oceania and both are flying species that occupy wetlands. *Gallirallus okinawae* (Okinawa rail; NC012140), is found endemic to wet forest on Okinawa island in the Japanese archipelago. *Gallirallus australis* (weka; KF701060) and *Porphyrio hochstetteri* (takahe; EF532934) endemic to New Zealand, and *Lewinia muelleri* (Auckland rail; KF644584) is endemic to the subantarctic Auckland Islands. *Gallirallus australis, P. hochstetteri* and *L. muelleri* live in mixed forest and grassland habitats. *Gallirallus okinawae, G. australis* and *P. hochstetteri* are absolutely flightless while *L. muelleri* is reported to fly well but infrequently.

### mtDNA genomes

Sample tissue details can be found in Table S1. Taking into account the already available mtDNA genomes of rails in GenBank we chose these species because of their geographical range in the Southern Hemisphere (e.g. *E. castaneoventris* and *L. muelleri*) and the relationships among genera. It has been inferred from molecular phylogenetics that Grues (suborder comprising Rallidae, Gruidae, Heliornithidae, Aramidae, Psophiidae and Aptornithidae) has a palaeo-austral signature [46], [49] but the fossil record is mainly found in the Northern Hemisphere. Although the intrafamiliar relationships in Rallidae are mostly unknown, we sought to include representatives of different and more distant genera in the family following Olson [42]. *Heliornis fulica* (sungrebe) was included as a close outgroup [36], [52]. We used a modified phenol-chloroform procedure [53] involving digestion in CTAB buffer for genomic DNA extraction. Genome DNA extractions were verified by gel electrophoresis and quantified using Qubit 2.0. An estimated 2–10 ng of each DNA was subjected to Whole Genome Amplification (WGA) via next generation sequencing (NGS) using the Illumina HiSeq platform (Beijing Genomics Institute, BGI) with 100 bp paired–end reads. Library preparation for sequencing was as described by Shendure and Ji [54] and Mardis [55].

### Sequence quality, mapping and assembly

For quality control of the fastq files we used FastQC v0.10.1 (http://www.bioinformatics.bbsrc.ac.uk/projects/fastqc) which helps to identify clusters with a low signal and low-quality base calls based on score value chastity ≥0.6. Contigs were created using *de novo* assembler Velvet v1.1.06 [56] which has been developed for assembly of short read using a Brujin graph algorithm. We conducted assemblies of the paired reads using multiple hash lengths (k = 43, 53, 63, 73, 83) and assembled the contigs obtained from the best kmer lengths (generally around 73). All the assemblies were performed on a server with 72 cores and 144 Gb access memory. Sequences were mapped using Geneious v6.0.5 [57] with reference to the previously published mtDNA genomes of Okinawa rail, GenBank accession number NC012140 [58] and common moorhen, GenBank accession number NC015236 [59], and visualized in Tablet v1.11.08.10 [60]. New mtDNA genomes were submitted to GenBank (Table S1).

### Phylogenetic analyses

For phylogenetic analyses, mtDNA genomes of additional Neognathae species were downloaded from GenBank. These lineages were from closely related groups to Rallidae (e.g. Gruidae, Otididae, Cuculidae) and provide appropriate context for dating analyses (Table S1). Galloanserae species were used as a known outgroup to all these taxa [36]. Several studies [36], [40], [61] have shown that the kagu (*Rhynochetos jubatus*) is not, despite some morphological and behavioural similarities, grouped within the Gruiformes; therefore this species was not included in the analyses.

Alignment of the mitochondrial sequences was performed with Geneious v6.0.5 [57] using manual adjustment. Each gene alignment was checked prior to phylogenetic analysis. We partitioned the aligned genomes into protein-coding genes, tRNAs, rRNAs, and noncoding fragments (including the origin of replication and the hypervariable region) [10]. We further partitioned the protein-coding genes based on amino acid sequences, into stems and loops data for rRNAs [62], [63] and cloverleaf pattern for tRNAs, which correspond to RNA secondary structures of those genes. Protein-coding genes were aligned manually based on the deduced amino acid sequences. The alignments of rRNA and tRNA genes were corrected by excluding ambiguous positions, such as loops and indels. Stop codons and ambiguous alignments next to gaps (conserved amino acid and RNA stems defined the inclusion boundaries of ambiguous regions next to gaps) were excluded from the alignment. The Control Region and NADH6 were excluded from the analyses due to alignment instability and heterogeneous base composition which can confound phylogenetic inferences.

The total length of the analysed mitogenomic dataset was 13,768 nucleotides which included the following partition scheme [10]: 1) first-codon position of the 12 protein-coding genes and 2) second-codon position of the 12 protein-coding genes, 3) RY–coding at the third-codon position of the 12 protein-coding genes, 4) loops of the tRNAs and rRNA combined, 5) stems of the tRNAs and rRNA combined. We performed all subsequent analyses with this partition strategy. Phylogenies were inferred using Bayesian Markov Chain Monte Carlo (MCMC) as implemented in MrBayes [64] with 20 million generations sampled every 2000 generations and a general time reversible model with gamma distribution (GTR+Γ) model of evolution. The model was estimated in ModelTest v3.7 using the Akaike Information Criterion [65]. Convergence and diagnostics of the Markov process were evaluated by the stability of parameter estimates across generations using Tracer v.1.6 (http://tree.bio.ed.ac.uk/software/tracer/). A burn in of 10% gave optimal results. We obtained Effective Sample Sizes (ESS) above 200 for all parameters. Maximum Likelihood (ML) with rapid bootstrapping was implemented in RAxML using GTR+Γ. Analyses were performed via the Cipres portal [66] and trees were viewed in FigTree v1.3.1 (http://tree.bio.ed.ac.uk/software/figtree/) and SplitsTree v4.12.8 [67].

### Time of divergences

Divergence times were estimated using a Lognormal relaxed Bayesian clock implemented in BEAST v1.7.5 [68]. A lognormal distribution was chosen because this shape accommodates greater flexibility regarding a cladogenetic event [69], [70]. For calibration constraints we used Galloanserae [71], [72] with a normal distribution of 66–86 Mya (95% range) and the stem sphenisciform *Waimanu* [38] with a normal distribution of 61.5–65.5 Mya (95% range). The *Waimanu* stem Penguin is without doubt the oldest specimen within the Sphenisciformes currently known [38], [73], [74] and *Vegavis*, the oldest reliable Galloanserae (anseriform) that is preserved in sufficient quality to provide contrasting anatomical characteristics with living birds [12], [72], [75]. These well-documented fossils provide confidence about their phylogenetic placement and serve together as phylogenetically external calibration points for estimation of divergence dates within neognaths and Gruiformes. They are considered to be reliable time constraints and have been widely used for divergence estimations because of the high confidence about their placement [69]. While there is some disagreement on the precise placement of *Vegavis* [76] within anseriforms, it demonstrates the existence of galloanserine birds in the late Cretaceous [76] and of extensive diversification and phylogenetically separated neognath lineages in the late Cretaceous/early Paleogene time [15], [16], [77].

We combined the results of three independent runs with 30 million generations and a burn-in of 10% for each. Chains were sampled every 5000^th^ generation and a Birth-Death process prior was used for the speciation model [78]. The analysis used a starting tree topology from the ML analysis modified with approximate years for branch lengths using TreeAnnotator [68]. We obtained for each run ESS above 200 for 98% of the parameters. The tree with the times of divergence and highest posterior density (HPD) intervals was visualized using FigTree v1.3.1 (http://tree.bio.ed.ac.uk/software/figtree/).

## Results

### Phylogenetic analyses

**Figure 2:**
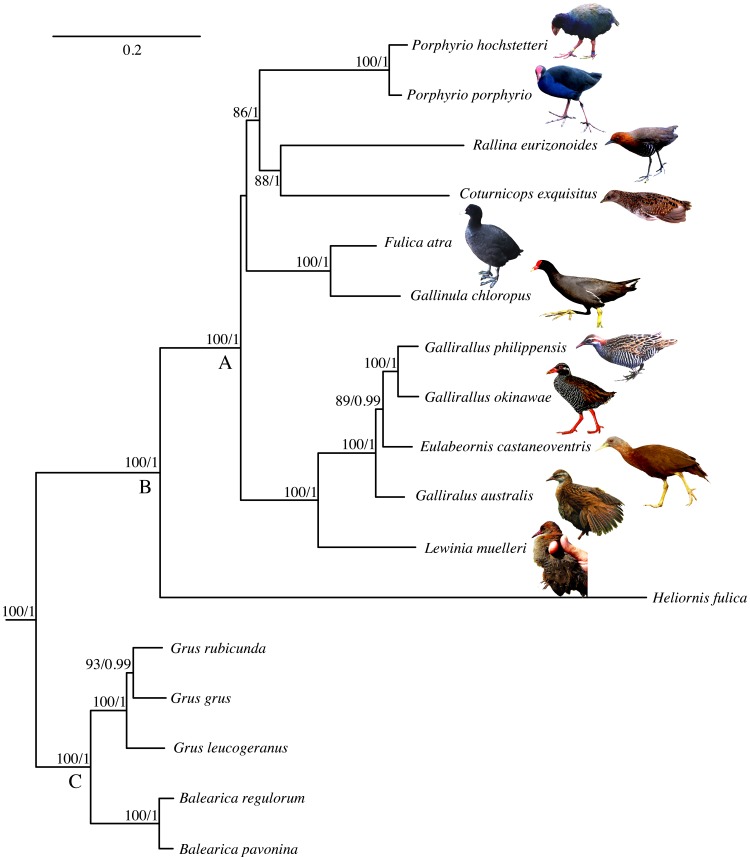
Maximum Likelihood tree resulting from analysis of complete mitochondrial genomes of birds with an emphasis in Rallidae. Species outside of the Grues are not shown. Bootstrap support over 70% and Bayesian posterior probabilities over 0.9 are indicated in each branch. Letters below the nodes refer to the families within the Order Gruiformes included in this study (A = Rallidae, B = Heliornithidae, C = Gruidae).

Inferred phylogenies and nodal support based on Bayesian and ML analyses were concordant and the tree topology (Figure 2) was congruent with trees previously inferred using nuclear and mitochondrial sequences [36], [46], [61]. In our analysis Rallidae was sister to Heliornithidae, and this clade in turn was sister to Gruidae [46]. We obtained similar levels of statistical support from ML and Bayesian analyses for internal groups within Rallidae with the expected pattern of intra–generic clusters (*Gallirallus* and *Porphyrio*). We found that *Eulabeornis castaneoventris* was placed within the genus *Gallirallus* and *G. australis* arises from the base of this clade [79], [80]. *Fulica atra* and *Gallinula chloropus* form a sister group, and *Coturnicops exquisitus* and *Rallina eurizonoides* form a sister clade to *Porphyrio*.

### Divergence times

**Figure 3:**
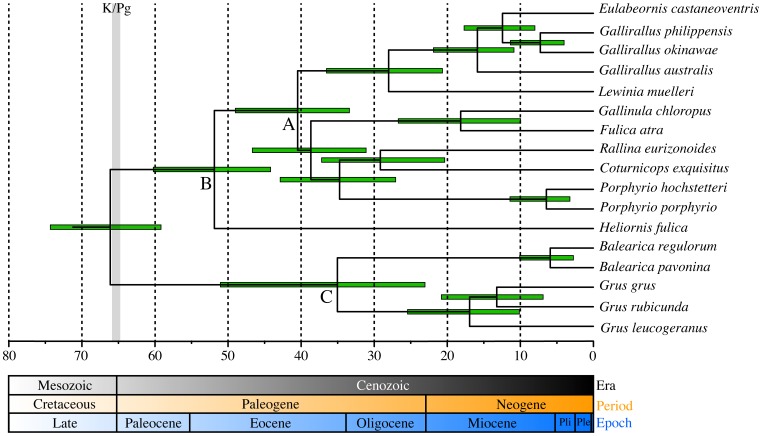
Chronogram based on analysis of complete mitochondrial genomes with a Lognormal relaxed–clock Bayesian analysis using BEAST. Age constraints were established by calibration fossils of Galloanserae with a minimum age of 66 Mya and maximum age of 86.5 Mya and Sphenisciformes with an age range from 61.5 Mya to 65.5 Mya. For each node the estimate time of divergence is indicate and the green bar represents the 95% HPD intervals of node ages. The time scale is in millions of years ago (Mya) and geological eras, periods and epochs are indicate where Pli is Pliocene and Ple is Pleistocene. A complete figure including all species analysed in this study is found in supplementary Figure S1. Bootstrap support, Bayesian posterior probabilities and letters referring families within order Gruiformes are the same as in Figure 2.

The long branch leading to *Heliornis fulica* (see Figure 2), might result from heterogeneity of mutation rates among lineages that can influence phylogenetic topology and time estimations. To allow for this possibility we estimated the times of divergence both with and without *H. fulica*, with similar results returned by Bayesian MCMC analyses. We report here the results including *H. fulica* (Figures 3 and S1). The split of Grues into rail–like and crane–like lineages is supported as occurring around the boundary of K–Pg mass extinction 66 (74–59) Mya [49]. The estimated time of divergence of the Heliornithidae and Rallidae (Ralloidea) lineages was 52 (60–44) Mya. Our divergence time analyses for crown group Rallidae is post K–Pg occurring during the Eocene about 40.5 (49–33) Mya. Among representatives of Rallidae included in this analysis there was evidence of several relatively early lineage-splitting events during the Late Eocene and Early Oligocene. Estimations of the divergence times obtained in this study indicate a common ancestor for *Gallirallus* and *Lewinia* around 28 (36–21) Mya, a divergence time of *Rallina* and *Coturnicops* around 29 (37–21) Mya, and origination of the aquatic *Fulica* and *Gallinula* clade 18 (26–10) Mya.

## Discussion

### Molecular phylogeny

Our phylogenetic analysis of bird mitogenomes is consistent with inferences made from several studies using fewer loci and provides strong corroboration of phylogenetic hypotheses and observed pattern of the molecular rate of evolution [36], [46]. The topology is consistent with that of Fain et al. [46], and relative branch lengths are similar with respect to the short internal branches in Gruidae and the comparatively long branches in Rallidae/Heliornithidae [36], [46]. The disparity in length of these branches is detected in both nuclear and mitochondrial data, suggesting it reflects real variation in the rates of molecular evolution among clades [46]. Rails contribute approximately 85% of the species in Grues diversity and this high level could potentially be attributable to a relatively fast mutation rate [81], [82]. The scarcity of heliornithids (three species) contradicts this idea but the disparity in species numbers could be due to nonuniform diversification rates through time and/or uneven extinction rates [83], [84].

### Origin and evolution of rails

Previous studies have addressed the question of the origin of the Gruiformes [46], [49] and the biogeography and evolution of some lineages of rails have been recently considered [47], [80], [85], [86]. Nevertheless, identification of the basal split and diversification of the rails has remained uncertain because of differences in the estimated dates and approaches used [46], [49]–[51].

We find the width of the 95% HPD intervals for Heliornithidae-Rallidae clade divergence and the crown-group Rallidae in our analysis overlap with interval age estimations reported from nuclear genes by Fain [46], Houde [49], and Brown [51]. However, the mean estimates of Heliornithidae-Rallidae divergence and basal Rallidae in the present analysis were much older than mean estimations in those studies (about twice the age). The observed tendency of analyses using mtDNA to overestimate node age compared to nuclear markers might be the source of this discrepancy [22], and it has been inferred that mtDNA data tends to result in overestimated ages of shallower nodes in particular [22], [70]. To minimise any potential overestimation of “shallower” nodes (compared to the nodes used to calibrate the tree) we applied several recommended strategies: 1) a partition scheme including RY–coding at the third-codon position; 2) relaxation of the molecular clock without assuming rate correlation among branches and; 3) variation across sites with GTR model and gamma distribution. We find that our estimations of “deeper” node ages (e.g. Ralloidea-Gruoidea divergence) are in fact very similar to those reported using nuclear data [49]. Although it appears that “shallower” node ages might be overestimates in our analysis (node A of the Rallidae lineage and node B of the Heliornithidae-Rallidae divergence in Figure 3), we have similar estimates for Gruinae-Balearicinae (node C in Figure 3) to other studies using nuclear [46] and mitochondrial data [87] and different calibration constraints. This suggests there is no systemic tendency for overestimation of late Eocene nodes. More probably, disparity with previous molecular analysis relates to difference in calibration constraints and taxon sampling of our study and others. For instance, Fain et al. [46] used as constraints external to Grues a lower (30 Mya) and upper (45 Mya) bound of the alcid-larid split.

Fossils of cranes (Gruidae) have been reported from the middle Eocene in Europe [88]–[90], and the earliest sungrebe fossil record is from the middle Miocene (14 Mya) in North America [91]. Two Paleogene Ralloidea fossils designated as *Messelornis* and *Walkbeckornis* are consistent with our estimated age of Ralloidea around 52 (60–44) Mya [15], [17]. The estimated time in our study for the common ancestor of living rallids is about 7 million years older than existing fossils assigned to the crown group. However, our lower interval value is consistent with European fossils within *Belgirallus* from the late Eocene–early Oligocene that have been suggested as representing the earliest Rallidae [92]–[94]. Recent examination of the humerus, coracoid and tarsometatarsus led to the proposal that *Belgirallus* belongs to stem group Ralloidea closely related to *Palaeoaramides* from the late Oligocene–early Miocene [52]. Nevertheless, great caution is needed in attribution of stem/crown group fossils when the availability of suitable comparisons is limited, systematics of the group is uncertain, and morphological characters can mislead phylogeny [35], [95]–[97]. The current absence of suitable fossils from the Eocene does not demonstrate that a common ancestor of living Rallidae did not exist at that time, and indeed some have been tentatively attributed to the family. *Palaeorallus, Eocrex* or *Fulicaletornis* from the Early Eocene in North America [94], [98] or rail–like taxa of the genus *Songzia* from the Early Eocene in China [99], [100] might represent extinct crown group rails, but their placement must remain equivocal [15], [88] because of the fragmentary nature of the specimens. The fossils at least hint that extinct species that share common ancestry with the living lineages in our analysis might have existed. As with the continuing discovery of Cretaceous bird fossils [12], [97], [101], [102], it is likely that better rail and Grues specimens will be forthcoming.

### Crown age of bird lineages

**Figure 4:**
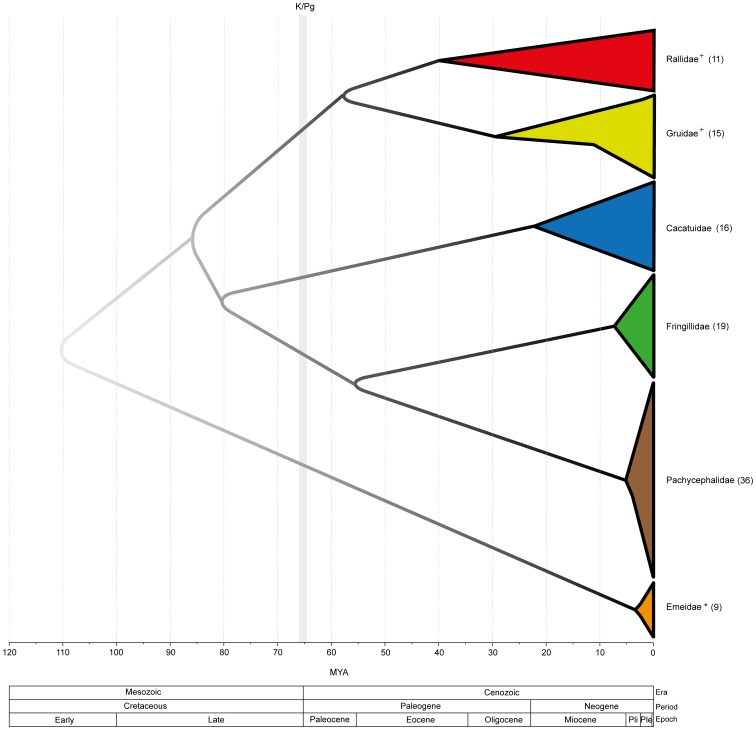
A schematic diagram representing available temporal patterns of diversification in bird lineages: Rallidae (this study), Gruidae [87], Cacatuidae [104], Fringillidae [103], Pachycephalidae [105] and Emeidae [32]. The number of species included in each study (in parenthesis) is represented by the respective clade height at zero time. Time of diversification among clades follows the most complete molecular time estimations on bird fauna [9]. Intensity of internal branch colour reflects the degree of confidence from available analyses that use various dating approaches. The cross symbol indicates studies using complete mitochondrial genomes to estimate divergence times. The asterisk indicates inclusion of Megalapterygidae and Dinornithidae species within Emeidae.

Studies of the origin and diversification of crown bird lineages provide insights into the rates and modes of ecological speciation. Comparisons of data from studies of birds makes it very clear that stem and crown group ages are not correlated, which is expected where speciation and extinction rates are uneven over time. For instance, several studies using complete mtDNA genomes or gene sequences show a relatively recent diversification of passerine and non–passerine bird lineages [32], [87], [103]–[105], with most crown lineages appearing during the Neogene (Figure 4), while taxa based on fossils assigned as part of stem groups are much older or younger than molecular date estimations [15], [34], [88], [106]–[108]. However, assessment of radiations in birds must be characterized by their geographical settings [9] because the spatial context of family level diversification is highly variable. For example, extant honeycreepers (family Fringillidae) in Hawaii [103] and whistlers (family Pachycephalidae) in the Indo-Pacific [105] represent recent insular radiations apparently responding to local ecological opportunities and climatic variations. Within the Palaeognaths, the extinct New Zealand moa radiation is classified in three families [32]. Available evidence indicates Pliocene diversification within Dinornithiformes; even if treated as a single New Zealand family, the moa clade shows a shallow insular radiation (Figure 4).

Insular lineages appear to have relatively shallow crown ages even though some archipelagos are comparatively old [109]–[111], whereas lineages that achieved wider distributions have deeper ages. For instance, widespread parrots (family Cacatuidae) [104], cranes (family Gruidae) [87] and rails (family Rallidae) have substantially deeper history (Figure 4). This indicates that a larger spatial range might increase the probability of lineage survival. The remarkable capacity of the rails to colonise and adapt to a wide variety of habitats perhaps favoured the retention of lineages through time. Rails show a fantastic capacity and propensity for range expansion and local adaptation with instances of supertramp species, such as *P. porphyrio* and *G. philippensis* [112], [113], which have colonized remote archipelagos in the Pacific [25]. However, the group has mainly retained a sedentary-ground walking ecology. Many lineages within Rallidae are not specialised to narrow marginal habitats but have proved resilient throughout the globe in diverse conditions. It seems likely that the temporal resilience of Rallidae and other cosmopolitan bird lineages has been guided by spatial and ecological plasticity. Further analysis with additional sampling will help reveal to what degree historical biogeographic signal has been retained in the current lineage distribution.

## Supporting Information

Figure S1 **Chronogram showing all species analysed in this study.** Divergence times are based on analysis of complete mitochondrial genomes with a relaxed-clock Bayesian analysis using BEAST. Bootstrap support over 70% and Bayesian posterior probabilities over 0.9 are indicated on each branch. Calibration constraints used to estimate divergence times are shown as red bars where a = calibration fossil of Galloanserae with a minimum age of 66 Mya and maximum age of 86.5 Mya, and b = calibration fossil of Sphenisciformes with an age range from 61.5 Mya to 65.5 Mya.
(TIF)

Table S1 **Taxa, Family and Order, museum voucher numbers, type of tissue, specimen sampling locality, GenBank accession numbers, and original source of data of the mtDNA genomes included in this study.** N/A = Not Available. Acronyms for museums are: ANWC = Australian National Wildlife Collection, Australia; MZUSP = Museu de Zoologia da Universidade de São Paulo (Brazil).
(RTF)
