# Supplementary figures and images for: Eocene Diversification of Crown Group Rails (Aves: Gruiformes: Rallidae)
Source: PLoS One. 2014 Oct 7;9(10):e109635. doi: 10.1371/journal.pone.0109635 (PMC4188725; doi:10.1371/journal.pone.0109635)

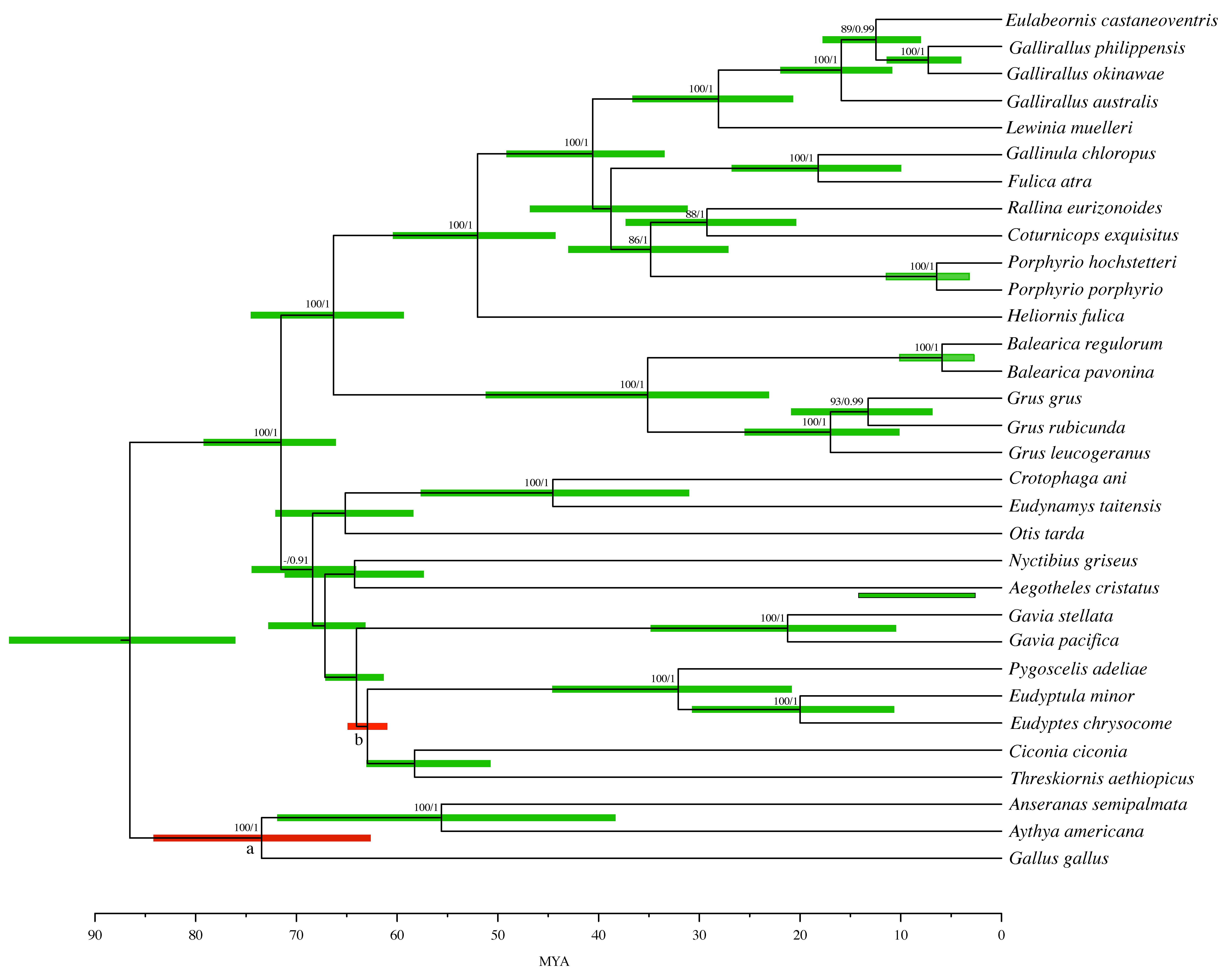

Supplement: Figure S1 — Chronogram showing all species analysed in this study. Divergence times are based on analysis of complete mitochondrial genomes with a relaxed-clock Bayesian analysis using BEAST. Bootstrap support over 70% and Bayesian posterior probabilities over 0.9 are indicated on each branch. Calibration constraints used to estimate divergence times are shown as red bars where a = calibration fossil of Galloanserae with a minimum age of 66 Mya and maximum age of 86.5 Mya, and b = calibration fossil of Sphenisciformes with an age range from 61.5 Mya to 65.5 Mya. (TIF) [file pone.0109635.s001.tif]
