# Supplementary material for: Eocene Diversification of Crown Group Rails (Aves: Gruiformes: Rallidae)
Source: PLoS One. 2014 Oct 7;9(10):e109635. doi: 10.1371/journal.pone.0109635 (PMC4188725; doi:10.1371/journal.pone.0109635)
Supplement: Table S1 — Taxa, Family and Order, museum voucher numbers, type of tissue, specimen sampling locality, GenBank accession numbers, and original source of data of the mtDNA genomes included in this study. N/A = Not Available. Acronyms for museums are: ANWC = Australian National Wildlife Collection, Australia; MZUSP = Museu de Zoologia da Universidade de São Paulo (Brazil). (RTF) [file pone.0109635.s002.rtf]

Table S1. Taxa, Family and Order, museum voucher numbers, type of tissue, specimen sampling locality, GenBank accession numbers, and original source of data of the mtDNA genomes included in this study. N/A = Not Available. Acronyms for museums are: ANWC = Australian National Wildlife Collection, Australia; MZUSP = Museu de Zoologia da Universidade de São Paulo (Brazil)
Species	Family/Order	Museum voucher	Type of tissue	Sample locality	GenBank ID	Source	
Coturnicops exquisitus 	Rallidae/Gruiformes	N/A	N/A	N/A	NC012143	Ozaki et al. 2010	
Eulabeornis castaneoventris	Rallidae/Gruiformes	ANWC50493	Muscle	Australia	KF644583	This study	
Fulica atra	Rallidae/Gruiformes	ANWC50980	Muscle	Australia	KF644582	This study	
Gallinula chloropus  	Rallidae/Gruiformes	N/A	N/A	N/A	HQ896036	Kan and Li, Unpublished	
Gallirallus australis	Rallidae/Gruiformes	N/A	Muscle	New Zealand	KF701060	This study	
Gallirallus okinawae  	Rallidae/Gruiformes	N/A	N/A	N/A	NC012140	Ozaki et al. 2010	
Gallirallus philippensis	Rallidae/Gruiformes	ANWC32326	Muscle	Australia	KF701061	This study	
Lewinia muelleri	Rallidae/Gruiformes	N/A	Blood	New Zealand	KF644584	This study	
Porphyrio hochstetteri 	Rallidae/Gruiformes	N/A	Muscle	New Zealand	EF532934	Morgan-Richards et al. 2008	
Porphyrio porphyrio	Rallidae/Gruiformes	N/A	Muscle	New Zealand	KF701062	This study	
Rallina eurizonoides sepiaria 	Rallidae/Gruiformes	N/A	N/A	N/A	NC012142	Ozaki et al. 2010	
Heliornis fulica	Heliornithidae/Gruiformes	MZUSP79862	Muscle	Brazil	KF644581	This study	
Balearica pavonina	Gruidae/Gruiformes	N/A	N/A	N/A	FJ769842	Krajewski et al. 2010	
Balearica regulorum	Gruidae/Gruiformes	N/A	N/A	N/A	FJ769841	Krajewski et al. 2010	
Grus grus 	Gruidae/Gruiformes	N/A	N/A	N/A	FJ769849	Krajewski et al. 2010	
Grus leucogeranus	Gruidae/Gruiformes	N/A	N/A	N/A	FJ769846	Krajewski et al. 2010	
Grus rubicunda	Gruidae/Gruiformes	N/A	N/A	N/A	FJ769853	Krajewski et al. 2010	
Otis tarda 	Otididae/Otidiformes	N/A	N/A	N/A	FJ751803	Yang et al. 2010	
Aegotheles cristatus	Aegothelidae/Caprimulgiformes	N/A	N/A	N/A	EU344979	Pratt et al. 2009	
Nyctibius griseus	Nyctibiidae/Caprimulgiformes	N/A	N/A	N/A	HM746792	Pacheco et al. 2011	
Crotophaga ani	Cuculidae/Cuculiformes	N/A	N/A	N/A	HM746794	Pacheco et al. 2011	
Eudynamys taitensis	Cuculidae/Cuculiformes	N/A	N/A	N/A	EU410487	Pratt et al. 2009	
Ciconia ciconia	Ciconiidae/Ciconiiformes	N/A	N/A	N/A	AB026818	Yamamoto, Unpublished	
Threskiornis aethiopicus	Threskiornithidae/Ciconiiformes	N/A	N/A	N/A	GQ358927	Cheng et al., Unpublished	
Eudyptes chrysocome	Spheniscidae/Sphenisciformes	N/A	N/A	N/A	AP009189	Watanabe et al. 2006	
Eudyptula minor	Spheniscidae/Sphenisciformes	N/A	N/A	N/A	AF362763	Slack et al. 2003	
Pygoscelis adeliae	Spheniscidae/Sphenisciformes	N/A	N/A	N/A	KC875855	Gibb et al. 2013	
Gavia pacifica	Gaviidae/Gaviiformes	N/A	N/A	N/A	AP009190	Watanabe et al. 2006	
Gavia stellata	Gaviidae/Gaviiformes	N/A	N/A	N/A	AY293618	Slack et al. 2006	
Aythya americana	Anatidae/Anseriformes	N/A	N/A	N/A	AF090337	Mindell et al. 1999	
Anseranas semipalmata	Anseranatidae/Anseriformes	N/A	N/A	N/A	AY309455	Harrison et al. 2004	
Gallus gallus	Phasianidae/Galliformes	N/A	N/A	N/A	AP003317	Nishibori 2003	
